# Polygenic Control of Carotid Atherosclerosis in a BALB/cJ × SM/J Intercross and a Combined Cross Involving Multiple Mouse Strains

**DOI:** 10.1534/g3.116.037879

**Published:** 2016-12-28

**Authors:** Andrew T. Grainger, Michael B. Jones, Mei-Hua Chen, Weibin Shi

**Affiliations:** * Department of Biochemistry and Molecular Genetics, University of Virginia, Charlottesville, Virginia 22908; † Radiology and Medical Imaging, University of Virginia, Charlottesville, Virginia 22908

**Keywords:** plaque, vessels, linkage mapping, haplotype analysis, dyslipidemia

## Abstract

Atherosclerosis in the carotid arteries is a major cause of ischemic stroke, which accounts for 85% of all stroke cases. Genetic factors contributing to carotid atherosclerosis remain poorly understood. The aim of this study was to identify chromosomal regions harboring genes contributing to carotid atherosclerosis in mice. From an intercross between BALB/cJ (BALB) and SM/J (SM) apolipoprotein E-deficient (*Apoe*^−/−^) mice, 228 female F2 mice were generated and fed a “Western” diet for 12 wk. Atherosclerotic lesion sizes in the left carotid artery were quantified. Across the entire genome, 149 genetic markers were genotyped. Quantitative trait locus (QTL) analysis revealed eight loci for carotid lesion sizes, located on chromosomes 1, 5, 12, 13, 15, 16, and 18. Combined cross-linkage analysis using data from this cross, and two previous F2 crosses derived from BALB, C57BL/6J and C3H/HeJ strains, identified five significant QTL on chromosomes 5, 9, 12, and 13, and nine suggestive QTL for carotid atherosclerosis. Of them, the QTL on chromosome 12 had a high LOD score of 9.95. Bioinformatic analysis prioritized *Arhgap5*, *Akap6*, *Mipol1*, *Clec14a*, *Fancm*, *Nin*, *Dact1*, *Rtn1*, and *Slc38a6* as probable candidate genes for this QTL. Atherosclerotic lesion sizes were significantly correlated with non-HDL cholesterol levels (*r* = 0.254; *p* = 0.00016) but inversely correlated with HDL cholesterol levels (*r* = −0.134; *p* = 0.049) in the current cross. Thus, we demonstrated the polygenic control of carotid atherosclerosis in mice. The correlations of carotid lesion sizes with non-HDL and HDL suggest that genetic factors exert effects on carotid atherosclerosis partially through modulation of lipoprotein homeostasis.

Stroke is the leading cause of extended disability and a major cause of mortality in the United States (Mozaffarian *et al.* 2015). 800,000 people are estimated to experience a new or recurrent stroke and 131,000 die of stroke annually in this country. Ischemic stroke accounts for ∼85% of all stroke cases and a large fraction of them are caused by atheromas in the carotid arteries (Donnan *et al.* 2008). Plaque in the carotid arteries directly or indirectly, though thrombus formation, blocks the blood flow to the brain (Markus and Cullinane. 2001; Matteis *et al.* 1999). Genetic studies of twins and families indicate that carotid arterial intima-media thickness and plaque, which reflect a thickening of the carotid artery wall and the presence of large irregular arterial wall deposits, respectively, is a genetically determined trait with heritability ranging from 30 to 65% (Sacco *et al.* 2009; Swan *et al.* 2003; Zhao *et al.* 2008). Recent genome-wide association studies (GWAS) have identified over a dozen common variants associated with carotid intima-media thickness and plaque, including LRIG1, EDNRA, SLC17A4, PIK3CG, PINX1, ZHX2, APOC1, LDLR, ANGPT1, ZBTB7C, HDAC9, the BCAR1-CFDP1-TMEM170A locus, EBF1, and PCDH15 (Bis *et al.* 2011; Du *et al.* 2015; Xie *et al.* 2015). However, these variants explain only a tiny fraction of the total heritability of the traits, suggesting that many more remain to be discovered. Furthermore, it is challenging to assess causality between a variant and disease in humans due to small gene effects, complex genetic structures, and environmental influences. Genetic studies of animal models have contributed greatly to the understanding of the genetic basis of human diseases, including atherosclerosis. *Apoe*^−/−^ mice develop all phases of atherosclerotic lesions in large- and medium-sized arteries, including the carotid arteries. QTL analysis for carotid atherosclerosis has been performed on two F2 populations derived from C57BL/6 (B6), C3H/HeJ (C3H), and BALB/cJ (BALB) strains and identified several significant and suggestive loci for the trait (Li *et al.* 2008; Rowlan *et al.* 2013a). Nevertheless, more crosses are needed to identify new QTL and expedite the finding of underlying genes for carotid atherosclerosis. We have recently found that *Apoe*^−/−^ mice with a SM/J (SM) genetic background developed significantly larger atherosclerotic lesions than those with a BALB background (Liu *et al.* 2015). In the present study, we generated a female F2 cohort from an intercross between the two *Apoe*^−/−^ strains to search for loci contributing to carotid atherosclerosis. The combined cross analysis using data from multiple intercrosses has been shown to improve the resolution of shared QTL and increase the power of identify new QTL not found in an individual cross (Li *et al.* 2005). Thus, in this study we also performed a combined cross-linkage analysis using data from the current cross and two previously reported B6 × C3H and B6 × BALB intercrosses (Li *et al.* 2008; Rowlan *et al.* 2013a).

## Materials and Methods

### Animals and experimental design

BALB and SM *Apoe*^−/−^ mice were generated in our laboratory using the classic congenic breeding strategy, as described by Liu *et al.* (2015) and Zhang *et al.* (2012). The two *Apoe*^−/−^ strains were crossed to generate F1s, which were intercrossed to generate a F2 population. Mice were weaned at 3 wk of age onto a chow diet. At 6 wk of age, female F2 mice were switched onto a Western diet containing 21% fat, 34.1% sucrose, 0.15% cholesterol, and 19.5% casein (TD 88137; Envigo) and maintained on the diet for 12 wk.

### Quantitation of carotid atherosclerosis

Atherosclerotic lesion sizes in the left common carotid artery and its main branches were measured as previously reported with minor modifications (Li *et al.* 2008). Briefly, the vasculature of mice was perfused through the heart with 4% paraformaldehyde, then the distal portion of the left common carotid artery and its adjacent branches were dissected *en bloc* and embedded in OCT compound (Tissue-Tek). Cryosections in 10 μm thickness were collected in every three sections, stained with oil red O and hematoxylin, and counterstained with fast green. Lesion areas were measured under a microscope using Zeiss AxioVision 4.8 software. Carotid lesion sizes on all sections were added up for each mouse and this sum was used for statistical analysis.

### Measurements of plasma lipids and glucose

Plasma total cholesterol, HDL cholesterol, triglyceride, and glucose were measured using assay kits as reported (Tian *et al.* 2005; Wang *et al.* 2015). Non-HDL cholesterol was calculated as the difference between total and HDL cholesterol.

### Genotyping

The Illumina mouse LD linkage panel consisting of 377 SNP loci was used to genotype F2 mice, as reported (Wang *et al.* 2015). Microsatellite markers were typed for chromosome 8 where only one SNP marker was informative. DNA from the two parental strains and F1s served as controls. After excluding uninformative and poorly typed SNPs, 149 markers were included in genome-wide QTL analysis.

### Statistical analysis

QTL analysis was performed using J/qtl. Genome-wide LOD score thresholds for significant or suggestive linkage were determined through 1000 permutations, as reported (Wang *et al.* 2015; Su *et al.* 2006; Yuan *et al.* 2009).

### Combined cross analysis

A combined cross analysis was performed using data from the current cross and two previously published B6 × C3H and B6 × BALB intercrosses (Li *et al.* 2008; Rowlan *et al.* 2013a). Genotype data for the chromosomal regions where a suggestive or significant QTL was found in an individual cross were recoded as “High” for F2s homozygous for the allele contributing to a larger lesion size, “Low” for F2s homozygous for the allele contributing to a smaller lesion size, and “H” for F2s with heterozygous alleles at each marker. For all other regions where no QTL was found, alleles at each marker were recoded based on the progenitor strain phenotype as reported (Wergedal *et al.* 2007). Phenotype data on carotid lesion sizes were switched from the total lesion area to the average of the top five lesion sizes for each F2 mouse in all crosses.

### Prioritization of candidate genes

Bioinformatic tools were used to prioritize candidate genes for major QTL that were mapped in two or more crosses derived from different parental strains. Probable candidate genes were those that contained one or more nonsynonymous SNPs or a SNP in the upstream regulatory region, and that SNP was shared by the progenitor strains carrying the high allele but different from the one shared by the progenitor strains carrying the low allele at a QTL, as reported (Rowlan *et al.* 2013b; Grainger *et al.* 2016).

### Data and reagent availability

BALB-Ape^−/−^ mice are available upon request. Supplemental Material, File S1 contains original genotype and phenotype data used for the current study. 

## Results

### Trait value frequency distribution

**Figure 1:**
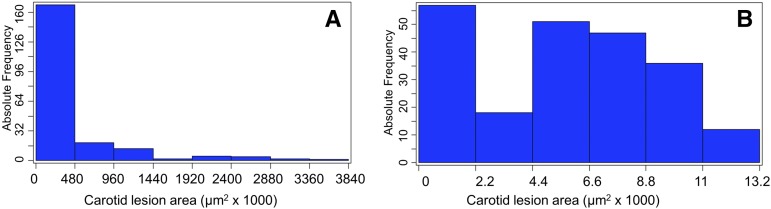
Frequency distributions of untransformed (A) and log2-transformed (B) total carotid lesion areas of 228 female F2 mice derived from BALB-*Apoe*^−/−^ and SM-*Apoe*^−/−^ mice. Each histogram indicates the number of individual F2 mice with a certain lesion area. *Apoe*^−/−^, apolipoprotein E-deficient.

Values of atherosclerotic lesion sizes in the left carotid arteries of 228 F_2_ mice were distributed in the Pareto manner: the frequency of F2 mice with a total lesion size of ≤ 480 × 1000 μm^2^ was the highest and then decreased with increasing lesion sizes (Figure 1). After being log2-transformed, these values exhibited a bimodal distribution with 25% of the F2 mice (*n* = 57) falling under the no or small lesion peak on the left (Log2 value < 2.2) and the remaining 75% falling under the bell-shaped curve on the right.

### QTL analysis of carotid lesion sizes

**Figure 2:**
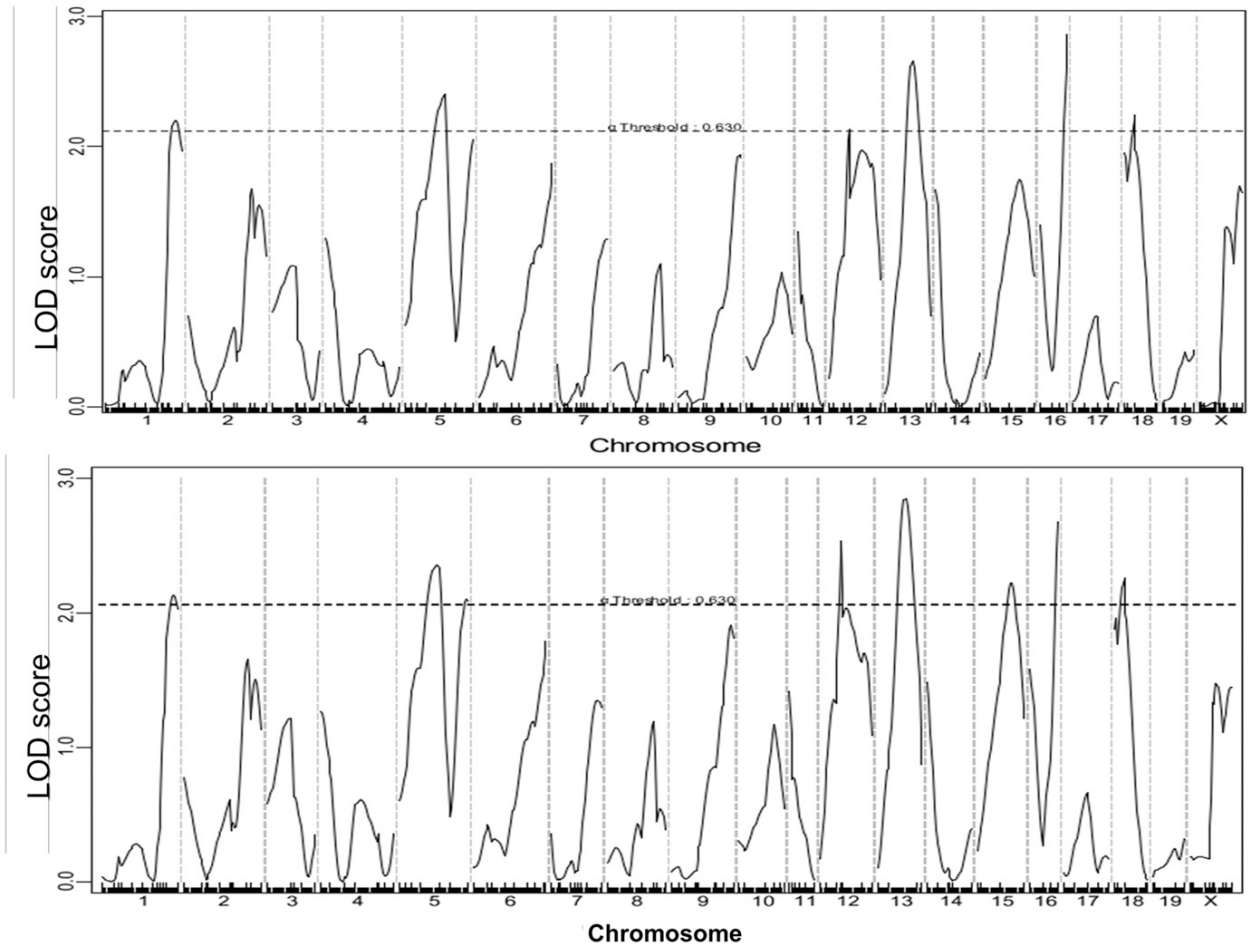
Genome-wide QTL analysis for carotid lesion sizes in the F2 population. Chromosomes 1 through 20 are represented numerically on the *x*-axis. *y*-axis represents LOD score. The horizontal dashed line denotes the genome-wide threshold for suggestive linkage, which was determined by 1000 permutations. Top panel: a genome-wide scan using untransformed carotid lesion data performed with the nonparametric algorism; bottom panel: a genome-wide scan using log2-transformed carotid lesion data performed with the parametric mode. LOD, logarithm of the odds; QTL, quantitative trait locus.

Genome-wide scans for carotid lesion sizes were performed using both a nonparametric algorithm to analyze nontransformed lesion data and a parametric algorithm to analyze Log2-transformed lesion data (Figure 2). Eight suggestive QTL, located on chromosomes 1, 5, 12, 13, 15, 16, and 18, were detected. With the exception of the QTL on distal chromosome 5 and the one on chromosome 15, which were only detected with the nonparametric algorithm, all QTL were detected on both scans (Table 1). The QTL on chromosome 12 peaked at 30.28 cM and had a LOD score of 2.48. This QTL replicated *Cath1*, a locus for carotid atherosclerosis originally mapped in the B6 × C3H *Apoe*^−/−^ intercross and then replicated in the B6 × BALB *Apoe*^−/−^ intercross (Li *et al.* 2008; Rowlan *et al.* 2013a). Two QTL on chromosome 5 were detected: the proximal one had a suggestive LOD score of 2.33 and peaked at 63.4 cM, and the distal one had a LOD score of 2.03 and peaked at 99.4 cM. The distal locus overlapped with *Cath2*, mapped initially in the B6 × C3H *Apoe*^−/−^ intercross as a suggestive QTL for carotid atherosclerosis and then replicated in the B6 × BALB *Apoe*^−/−^ intercross as a highly significant QTL (Li *et al.* 2008; Rowlan *et al.* 2013a). The locus on chromosome 13 peaked at 34.02 cM and had a suggestive LOD score of 2.8. This QTL replicated *Cath3*, mapped in the B6 × BALB *Apoe*^−/−^ intercross (Rowlan *et al.* 2013a). The QTL on chromosome 15 peaked at 46.74 cM and had a suggestive LOD score of 2.24. This QTL overlapped with a suggestive locus for atherosclerosis in the innominate artery and mapped a B6 × C3H *Apoe*^−/−^ intercross (Bennett *et al.* 2015). We named it *Cath5* as this QTL was mapped in two separate crosses. The QTL on chromosome 18 had a suggestive LOD score of 2.22 and peaked at 16.27 cM. It replicated a suggestive QTL for carotid atherosclerosis mapped in the B6 × BALB *Apoe*^−/−^ intercross (Rowlan *et al.* 2013a), and was named *Cath6*.

**Table 1:** QTL identified for carotid lesion areas in female F2 mice derived from an intercross between BALB-*Apoe*^−/−^ and SM-*Apoe*^−/−^ mice

Locus	Chr	Analysis	LOD*^a^*	*p*-Value*^b^*	Peak (cM)	95% C.I.*^c^*	High Allele	Mode of Inheritance*^d^*
	1	Nonparametric	2.17	0.535	91.52	75.52–97.02	—	Heterosis
	5	Nonparametric	2.33	0.422	63.4	34.19–101.24	BALB	Additive
*Cath2*	5	Nonparametric	2.03	0.630	99.4	79.4–101.2	BALB	Dominant
*Cath1*	12	Nonparametric	2.48	0.324	30.28	19.41–63.41	SM	Additive
*Cath3*	13	Nonparametric	2.8	0.163	34.02	22.02–46.02	SM	Dominant
*Cath5*	15	Nonparametric	2.24	0.474	46.74	26.74–62.74	SM	Recessive
	16	Nonparametric	2.58	0.274	44.66	13.43–46.66	BALB	Dominant
*Cath6*	18	Nonparametric	2.22	0.497	16.27	3.73–27.73	SM	Additive
	1	Parametric	2.23	0.545	87.52	77.52–97.02	—	Heterosis
	5	Parametric	2.38	0.413	67.27	33.4–101.4	BALB	Additive
*Cath2*	5	Parametric	2.03	0.630	99.4	79.4–101.2	BALB	Additive
*Cath1*	12	Parametric	2.1	0.644	30.28	23.41–65.41	SM	Additive
*Cath3*	13	Parametric	2.64	0.267	32.02	22.02–47.99	SM	Dominant
	16	Parametric	2.81	0.205	46.66	13.43–46.66	BALB	Dominant
*Cath6*	18	Parametric	2.21	0.552	16.27	3.73–25.73	SM	Additive

Chr, chromosome; LOD, logarithm of the odds; QTL, quantitative trait locus.

a LOD scores were obtained from genome-wide scans using J/qtl. LOD score threshold for suggestive QTL > 2.054; for significance > 3.314 established by 1000 permutation tests.

b *p*-values represent genome-wide significance at each locus.

c 95% C.I. was determined through whole-genome scans.

d Inheritance was determined based on the effect of each parental allele at the nearest genomic marker.

The QTL on chromosome 1 peaked at 91.52 cM and had a LOD score of 2.17. It overlapped with *Ath1*, a QTL for aortic atherosclerosis mapped in a number of crosses (Wang *et al.* 2005; Grainger *et al.* 2016; Zhang *et al.* 2012). The QTL on chromosome 16 peaked at 46.66 cM and had a score of 2.58, and this QTL was novel. The SM allele was associated with increased lesion sizes for chromosome 12, 13, 15, and 18 QTL, while the BALB allele was associated with increased lesion sizes for the chromosome 5 and 16 QTL (Table 2). The chromosome 1 QTL affected lesion formation in a heterotic manner in that F2 mice with heterozygous alleles exhibited increased lesion size over those with homozygous alleles.

**Table 2:** Effects of BALB and SM alleles on carotid lesion area at identified QTL in female F_2_ mice derived from BALB-*Apoe*^−/−^ and SM-*Apoe*^−/−^ mice

Locus Name	Chr	Analysis	Peak Marker	Peak (cM)	BB	BS	SS	*p*-Value
	1	Nonparametric	rs3685643	91.52	281.5 ± 662.4	522.8 ± 1049.5	260.9 ± 466.9	0.016
	5	Nonparametric	rs3726547	63.4	604.1 ± 1250.9	341.5 ± 710.1	273.3 ± 504.0	0.006953
*Cath2*	5	Nonparametric	rs13478578	99.4	454.6 ± 629.3	412.1 ± 1023.7	285.8 ± 635.7	0.008146
*Cath1*	12	Nonparametric	rs13481509	30.28	171.1 ± 389.1	374.9 ± 952.2	652.9 ± 917.3	0.002917
*Cath3*	13	Nonparametric	rs6259014	34.02	313.9 ± 650.0	412.7 ± 938.2	427.7 ± 793.2	0.143
*Cath5*	15	Nonparametric	rs13482641	46.74	244.8 ± 397.0	294.2 ± 664.1	711.2 ± 1281.3	0.03
	16	Nonparametric	rs3721202	44.66	426.5 ± 1249.9	485.8 ± 192.8	192.8 ± 405.0	0.002091
*Cath6*	18	Nonparametric	rs3683699	16.27	256.1 ± 440.2	423.9 ± 1059.8	539.0 ± 759.6	0.005427
	1	Parametric	rs3685643	87.52	4.3 ± 3.8	6.1 ± 3.7	5.8 ± 3.2	0.01199282
	5	Parametric	rs3726547	67.27	6.8 ± 3.3	5.2 ± 3.6	4.8 ± 3.8	0.00624255
*Cath2*	5	Parametric	rs13478578	99.4	6.4 ± 3.6	5.6 ± 3.5	4.2 ± 3.8	0.00941731
*Cath1*	12	Parametric	rs13481509	30.28	4.7 ± 3.2	5.3 ± 3.8	6.8 ± 3.8	0.00787988
*Cath3*	13	Parametric	rs6259014	32.02	4.6 ± 3.8	5.7 ± 3.7	6.0 ± 3.6	0.14731571
	16	Parametric	rs3721202	46.66	5.8 ± 3.4	6.2 ± 3.7	4.0 ± 3.6	0.00150063
*Cath6*	18	Parametric	rs3683699	16.27	5.3 ± 3.5	5.0 ± 3.9	7.1 ± 3.2	0.00613849

Measurements for carotid lesion areas are expressed as means ± SD. The unit for these measurements is: µm^2^ × 1000 for nonparametric analysis. For parametric analysis, the values are log2-transformed total carotid lesion areas. The Kruskal–Wallis test was used on the nonparametric data and ANOVA on the parametric data to determine the significance (*p*-value) of the differences among the BB, BS, and SS genotypes. Chr, chromosome; BB, homozygous for the BALB allele at the linked peak marker; BS, heterozygous for both BALB and SMJ; SS, homozygous for the SMJ allele.

### Combined cross analysis for overlapping QTL

**Figure 3:**
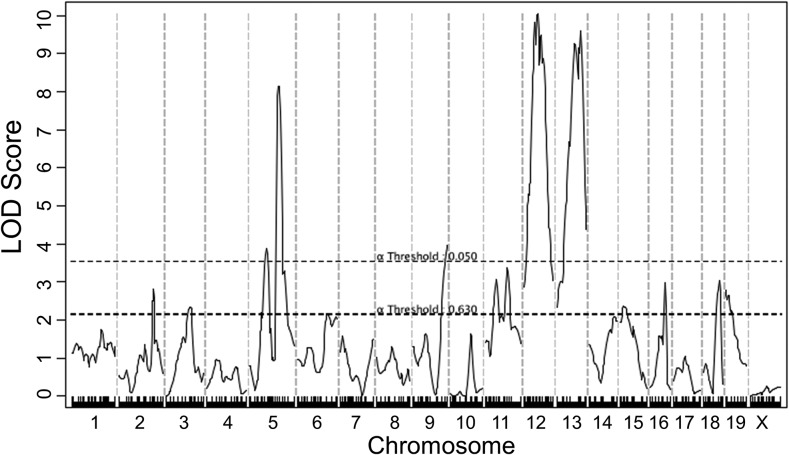
Genome-wide QTL analysis for carotid lesion sizes using combined data from the current cross and two previously reported B6 × BALB and B6 × C3H *Apoe*^−/−^ intercrosses. The horizontal dotted lines indicate the thresholds for genome-wide suggestive and significant linkage, as determined by 1000 permutations. *Apoe*^−/−^, apolipoprotein E-deficient; LOD, logarithm of the odds; QTL, quantitative trait locus.

**Figure 4:**
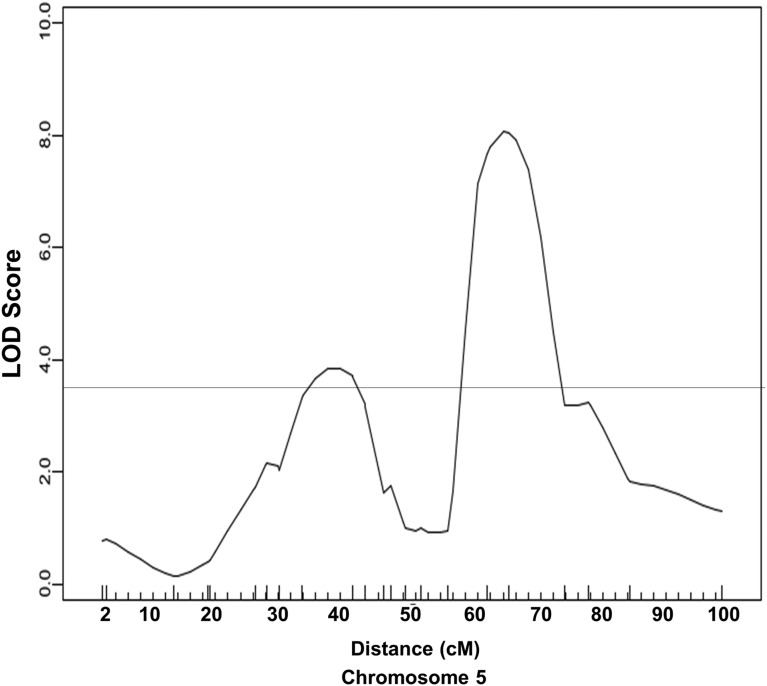
Interval mapping graph for carotid lesion size on chromosome 5 using combined data from the current cross and previously reported B6 × BALB and B6 × C3H *Apoe*^−/−^ intercrosses. The horizontal line denotes the threshold for significant linkage. *Apoe*^−/−^, apolipoprotein E-deficient; LOD, logarithm of the odds.

Combined cross analysis was performed for carotid atherosclerosis using data from the current cross and two previously reported B6 × C3H and B6 × BALB intercrosses (Li *et al.* 2008; Rowlan *et al.* 2013a). Five significant QTL, located on chromosomes 5, 9, 12, and 13, and nine suggestive QTL on chromosomes 2, 3, 6, 11, 15, 16, 18, and 19, were identified (Figure 3 and Table 3). The majority of these QTL had been identified as significant or suggestive QTL in one or more individual crosses, but the LOD scores for the significant QTL on chromosomes 5, 9, 12, and 13 were higher compared to those determined in an individual cross. The 95% C.I. was relatively smaller than that in an individual cross for most QTL. A LOD score plot for chromosome 5 revealed two disparate peaks, indicating the presence of two QTL for carotid atherosclerosis (Figure 4). The distal QTL replicated *Cath2*, mapped in all the three crosses (Li *et al.* 2008; Rowlan *et al.* 2013a). The proximal QTL was visible as a distinct peak in the current cross as well as the previously reported B6 × BALB intercross (Rowlan *et al.* 2013a), and was named *Cath7* to represent a new locus for carotid atherosclerosis. The significant QTL on chromosome 9 was initially mapped as a suggestive QTL in the B6 × BALB intercross (Rowlan *et al.* 2013a), and was named *Cath8*. The suggestive QTL on chromosomes 6, 11, 15, 16, and 18 were each mapped in one or more individual crosses, while the suggestive QTL on chromosomes 2, 3, and 19 were only detected in the combined cross.

**Table 3:** Significant and suggestive QTL for carotid atherosclerosis identified in combined cross analysis of data from the current cross and the two previously reported crosses

Locus	Chr	Trait	LOD	Peak (cM)	95% C.I.	Peak (Mb)	95% C.I. (Mb)
	2	Carotid lesion	2.77	80.22	44.22–98.22	159.59	71.96–170.59
	3	Carotid lesion	2.31	50.01	26.01–64.01	114.85	56.96–138.77
***Cath7***	**5**	**Carotid lesion**	**3.84**	**39.05**	**34.19**–**44.28**	**65.31**	**61.51**–**69.16**
***Cath2***	**5**	**Carotid lesion**	**8.06**	**66.35**	**63.84**–**70.35**	**127.32**	**124.83**–**131.29**
*Cath4*	6	Carotid lesion	2.15	66.21	1.53–88.79	120.60	6.44–145.75
***Cath8***	**9**	**Carotid lesion**	**3.92**	**75.33**	**66.37**–**75.33**	**114.09**	**103.61**–**114.09**
	11	Carotid lesion	3.02	26.1	18.2–32.2	45.28	30.91–54.19
	11	Carotid lesion	3.32	51	17.99–69.99	83.84	30.91–105.15
***Cath1***	**12**	**Carotid lesion**	**9.95**	**32.59**	**23.47**–**44.59**	**70.23**	**48.06**–**88.56**
***Cath3***	**13**	**Carotid lesion**	**9.49**	**53.35**	**36.02**–**56.02**	**100.5**	**68.40**–**103.48**
*Cath5*	15	Carotid lesion	2.35	11.26	3.8–37.8	30.76	7.87–71.92
	16	Carotid lesion	2.94	41.66	28.95–43.66	64.11	37.35–72.76
*Cath6*	18	Carotid lesion	3.01	39.73	31.73–41.73	62.50	56.27–65.17
	19	Carotid lesion	2.74	2.43	2.43–26.43	3.65	3.65–36.64

LOD score threshold for suggestive QTL was 2.128 and was 3.508 for significant QTL. Significant QTL are highlighted in bold. Chr, chromosome; LOD, logarithm of the odds; QTL, quantitative trait loci.

### Candidate genes for Cath1

*Cath1* on chromosome 12 was mapped in the current cross and two previously reported B6 × C3H and B6 × BALB *Apoe*^−/−^ intercrosses (Li *et al.* 2008; Rowlan *et al.* 2013a). For this QTL, the B6 and SM alleles were associated with increased lesion sizes, while the C3H and BALB alleles were associated with smaller lesion sizes. We used the Sanger SNP database to search for positional candidate genes that contain nonsynonymous SNP(s) or SNP(s) in upstream regulatory regions that are shared by the low allele strains (BALB and C3H) but are different from ones carried by the high allele strain (B6) under the linkage peak. The SM strain was not included due to its incomplete genomic sequences for the region. Twenty-four candidate genes were identified (Table 4). Among them, *Eapp*, *Foxa1*, *Fancm*, *Nin*, *Dact1*, *Rtn1*, and *Trmt5* contained one or more nonsynonymous SNPs with a low SIFT (Sorting Intolerant From Tolerant) score, predicting a high likelihood that an amino acid substitution has an adverse effect on protein function.

**Table 4:** Haplotype analysis for *Cath1* on chromosome 12 (52–75 Mb)

Chr	Position	Gene	dbSNP	High Allele	Low Allele		Consequence	Amino Acid Change	SIFT Score	Tolerated
				C57BL/6	BALB_cJ	C3H_HeH				
12	52006466	Dtd2	rs46701436	A	G	G	Missense variant	Cn 7:V/A	0.92	Yes
12	52023971	Gpr33	rs29173669	A	G	G	Missense variant	Cn 95:V/A	0.71	Yes
12	52027979	Gpr33	rs51561875	T	G	G	5ʹ-UTR variant
12	52027989	Gpr33	rs49936313	T	A	A	5ʹ-UTR variant
12	52027993	Gpr33	rs47019843	C	T	T	5ʹ-UTR variant
12	52519522	Arhgap5	rs29198609	T	C	C	Missense variant	Cn 1092:V/A	1	Yes
12	52887261	Akap6	rs29183247	G	A	A	Missense variant	Cn 512:R/Q	0.2	Yes
12	52887389	Akap6	rs29223294	A	G	G	Missense variant	Cn 555:T/A	0.47	Yes
12	53140291	Akap6	rs48484112	G	A	A	Missense variant	Cn 1496:R/H	1	Yes
12	54203369	Egln3	rs29130898	A	G	G	Missense variant	Cn 65:C/R	0.89	Yes
12	54203615	Egln3	rs29122127	T	G	G	5ʹ-UTR variant
12	54203690	Egln3	rs13473456	G	A	A	5ʹ-UTR variant
12	54695720	Eapp	rs29183105	G	A	A	Missense variant	Cn 22:A/V	0.01	No
12	54941453	Baz1a	rs29195192	G	A	A	Missense variant	Cn 88:L/F	0.04	Yes
12	54999084	Baz1a	rs29196908	G	C	C	5ʹ-UTR variant
12	57303392	Mipol1	rs29163022	G	A	A	5ʹ-UTR variant
12	57325598	Mipol1	rs46300008	A	G	G	Missense variant	Cn 148:K/E	1	Yes
12	57325623	Mipol1	rs13481473	A	G	G	Missense variant	Cn 156:H/R	1	Yes
12	57542267	Foxa1	rs13481474	T	C	C	Missense variant	Cn 389:H/R	0	No
12	57576142	Ttc6	rs50478178	G	C	C	Missense variant	Cn 109:R/P	1	Yes
12	57725789	Ttc6	rs48534883	T	C	C	Splice region variant
12	58267790	Clec14a	rs31966428	T	C	C	Missense variant	Cn 349:I/V	0.23	Yes
12	58268339	Clec14a	rs13465063	T	C	C	Missense variant	Cn 166:T/A	1	Yes
12	58268988	Clec14a	rs29162388	C	G	G	5ʹ-UTR variant
12	58268992	Clec14a	rs29194398	G	C	C	5ʹ-UTR variant
12	64471729	Fscb	rs13481500	G	A	A	Missense variant	Cn 988:P/S	1	Yes
12	64472091	Fscb	rs29131205	G	C	C	Missense variant	Cn 867:A/G	0.21	Yes
12	64472965	Fscb	rs585463036	C	A	A	Missense variant
12	64473313	Fscb	rs29220106	G	A	A	Missense variant	Cn 460:P/S	0.2	Yes
12	64950146	Klhl28	rs33846378	C	T	T	Missense variant	Cn 474:V/I	0.07	Yes
12	65113969	Fancm	rs212043559	A	T	T	Missense variant	Cn 1407:N/I	0	No
12	65130342	Fancm	rs29212900	A	C	C	Missense variant	Cn 1987:I/L	0.47	Yes
12	65130397	Fancm	rs29213465	A	T	T	Missense variant	Cn 2005:Q/L	1	Yes
12	65130436	Fancm	rs29184120	A	C	C	Missense variant	Cn 2018:K/T	0	No (low confidence)
12	65149007	Mis18bp1	rs50634267	C	T	T	Missense variant	Cn 661: R/Q	0.27	Yes
12	65152837	Mis18bp1	rs29200949	T	C	C	Splice region variant
12	65172467	Mis18bp1	rs3695606	T	A	A	5ʹ-UTR variant
12	65172551	Mis18bp1	rs3696207	A	G	G	5ʹ-UTR variant
12	69204274	Pole2	rs3704977	T	C	C	Splice region variant
12	69223117	Pole2	rs29135637	T	C	C	Missense variant	Cn 78:M/V	0.43	Yes
12	69741794	Atp5s	rs29193315	G	A	A	Missense variant	Cn 156: V/I	1	Yes
**12**	**70043177**	**Nin**	**rs32225358**	**C**	**T**	**T**	**Missense variant**	**Cn 1155:E/K**	**0.06**	**Yes**
**12**	**70043386**	**Nin**	**rs29192398**	**C**	**T**	**T**	**Missense variant**	**Cn 1085:R/Q**	**0.01**	**No**
**12**	**70043389**	**Nin**	**rs29159683**	**G**	**T**	**T**	**Missense variant**	**Cn 1084:S/Y**	**0.02**	**No**
**12**	**70043915**	**Nin**	**rs29149025**	**T**	**C**	**C**	**Missense variant**	**Cn 909:K/E**	**1**	**Yes**
12	70180988	Abhd12b	rs29173916	G	T*	T*	Missense variant	Cn 258:M/I	1	Yes
12	70183081	Abhd12b	rs51691757	A	G	G	Splice region variant
12	70183205	Abhd12b	rs32247424	A	G*	G*	Stop retained variant, 3ʹ-UTR variant
12	70193813	Pygl	rs32246688	G	T	T	Splice region variant
12	70197551	Pygl	rs29151561	A	G	G	Splice region variant
12	70201877	Pygl	rs13467444	T	C	C	Missense variant	Cn 323:M/V	1	Yes
12	70231391	Pygl	rs45983203	C	T	T	5ʹ-UTR variant
12	70231392	Pygl	rs48603304	T	A	A	5ʹ-UTR variant
12	70231439	Pygl	rs50231886	A	T	T	5ʹ-UTR variant
12	70231450	Pygl	rs32251907	A	T	T	5ʹ-UTR variant
**12**	**71318068**	**Dact1**	**rs29185339**	**C**	**T**	**T**	**Missense variant**	**Cn 504:P/L**	**0.02**	**No**
**12**	**71318500**	**Dact1**	**rs29222974**	**G**	**C**	**C**	**Missense variant**	**Cn 648:R/P**	**0.44**	**Yes**
**12**	**72408325**	**Rtn1**	**rs3695552**	**T**	**C**	**C**	**Missense variant**	**Cn 76:E/G**	**0**	**No**
**12**	**72408926**	**Rtn1**	**rs29209324**	**T**	**C**	**C**	**5ʹ-UTR variant**
12	72454073	Lrrc9	rs29198846	G	A	A	Missense variant	Cn 191:R/H	0.97	Yes
12	73281229	Trmt5	rs29130757	G	T	T	Missense variant	Cn 400:P/H	0	No
12	73285238	Trmt5	rs29166240	A	T	T	Missense variant	Cn 15:L/M	0.15	No (low confidence)
12	73285259	Trmt5	rs29162033	A	T	T	Missense variant	Cn 8:F/I	0.06	Yes (low confidence)
12	73285271	Trmt5	rs29141846	C	A	A	Missense variant	Cn 4:V/L	1	Yes
12	73287081	Slc38a6	rs48749977	T	C	C	5ʹ-UTR variant
12	73350619	Slc38a6	rs13481528	C	T	T	Missense variant	Cn 345:A/V	1	Yes

Functional candidate genes are denoted in bold. A smaller SIFT score denotes a higher likelihood of protein function change. Chr, chromosome; dbSNP, Single Nucleotide Polymorphism Database identifier; SIFT, Sorting Intolerant From Tolerant; Cn, Coding non-synonymous polymorphism; UTR, untranslated region.

### Relationships of carotid atherosclerosis with plasma lipids and glucose

**Figure 5:**
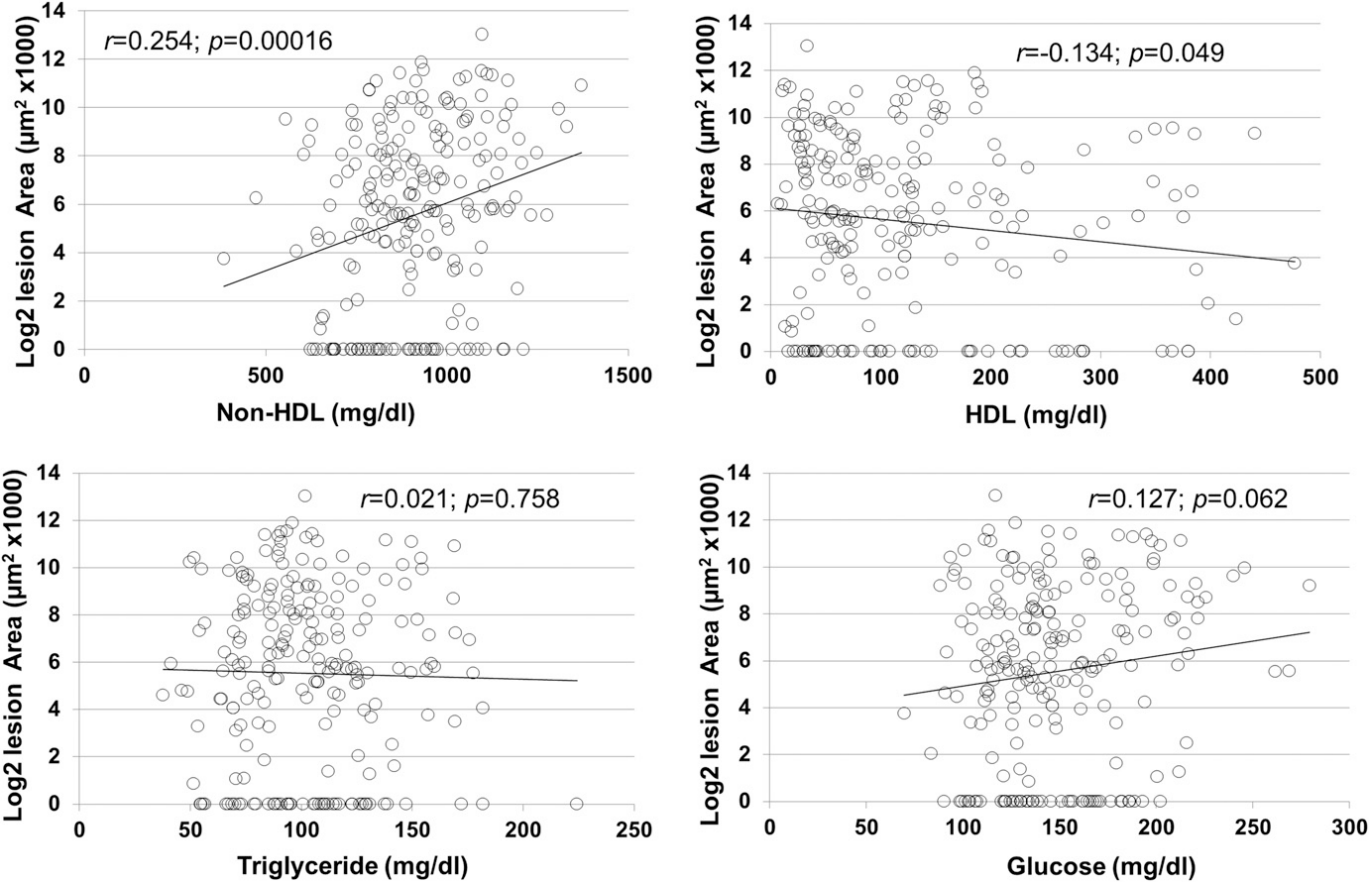
Scatterplots showing the correlations of carotid lesion sizes with plasma non-HDL (A), HDL cholesterol (B), triglyceride (C), and glucose (D) in the F2 population. Each point represents an individual value of a F2 mouse. The correlation coefficient (*r*) and significance (*p*) are shown. Log2-transformed carotid total areas were used for the analyses. HDL, high-density lipoprotein.

Associations of carotid lesion sizes with plasma HDL, non-HDL cholesterol, triglyceride, and glucose levels were evaluated using the F2 population (Figure 5). A significant correlation with non-HDL cholesterol levels was observed (*r* = 0.254; *p* = 0.00016). F2 mice with higher non-HDL cholesterol levels tended to develop larger carotid lesions. The value of the correlation coefficient *r*^2^ indicates that non-HDL accounted for 6.45% of the variance in carotid lesion sizes among the F2 population. A marginal, but statistically significant, inverse correlation with HDL cholesterol levels was observed (*r* = −0.134; *p* = 0.049). F2 mice with higher HDL cholesterol levels tended to develop smaller carotid lesions. HDL accounted for 1.8% of the variance in lesion sizes of the F2 mice. No correlation with triglyceride levels was found (*r* = 0.021; *p* = 0.758). There was a trend toward a significant correlation with plasma glucose levels (*r* = 0.127; *p* = 0.062).

## Discussion

Genetic factors contributing to carotid atherosclerosis, which is a major cause of ischemic stroke, are poorly understood. In this study, we performed QTL analysis using data from a newly generated intercross and combined data from three independent intercrosses to search for QTL contributing to carotid atherosclerosis. Five significant QTL and > 10 suggestive QTL were identified for the trait. Bioinformatic tools were successfully used to reduce the number of candidate genes for *Cath1*. Moreover, plasma non-HDL cholesterol was found to explain 6.5% of the variance in carotid lesion sizes of the F2 population.

Atherosclerotic lesions in the left carotid artery were measured after F2 mice were fed a Western diet for 12 wk. Under this condition, these mice, which were on the *Apoe*-null background, developed severe hyperlipidemia (Wang *et al.* 2015). Nevertheless, we found that a large fraction of F2 mice developed little or no atherosclerotic lesions in the carotid artery. The same phenomenon has also been observed in two other intercrosses previously constructed for QTL analysis of carotid atherosclerosis in the mouse (Li *et al.* 2008; Rowlan *et al.* 2013a). In contrast, all the F2 mice developed atherosclerotic lesions in the aortic root (Grainger *et al.* 2016). As the aortic root and the carotid arteries are exposed to the same level of lipoproteins and the same type of blood cells, the site-specific difference in the development of atherosclerosis should be attributable to local factors, such as vascular geometry, blood flow dynamics, and vessel wall properties. A genetic study of aortic arch curvature and atherosclerosis in a mouse cross has linked genetic factors regulating aortic arch geometry to aortic lesion formation (Tomita *et al.* 2010).

We and others have found that QTL identified for atherosclerotic lesions in the aortic root can be quite different from those mapped in another site of the vasculature, even in the same crosses (Li *et al.* 2008; Zhang *et al.* 2012; Rowlan *et al.* 2013a; Grainger *et al.* 2016; Bennett *et al.* 2015). Because the aortic root is easy to study in mice, genetic studies of atherosclerosis have largely focused on this site. However, this site has little clinical significance to humans. In contrast, the carotid arteries are the most extensively studied vessels in humans with ultrasonography because of their close association with the brain and ready accessibility.

*Cath1* on chromosome 12, *Cath2* on chromosome 5, *Cath3* on chromosome 13, and *Cath4* on chromosome 6 are four significant QTL for carotid atherosclerosis thus far mapped in two *Apoe*^−/−^ mouse intercrosses (Li *et al.* 2008; Rowlan *et al.* 2013a). Three of the four QTL were replicated in the current BALB × SM *Apoe*^−/−^ intercross, and all of them were replicated in the combined cross analysis. The QTL on chromosome 15 overlapped in the C.I. with a suggestive locus affecting both atherosclerotic lesion size and composition in the innominate artery of *Apoe*^−/−^ mice (Bennett *et al.* 2009). We named it *Cath5* to represent a locus for carotid atherosclerosis in the mouse. Naming a suggestive locus is considered appropriate if it is repeatedly observed (Abiola *et al.* 2003). The QTL on chromosome 18 overlapped in the C.I. with a suggestive locus for carotid atherosclerosis mapped in the B6 × BALB *Apoe*^−/−^ intercross, and was named *Cath6*.

Five significant QTL and nine suggestive QTL for carotid atherosclerosis were identified in the combined cross analysis. Nearly all of these QTL were mapped in one or more individual crosses. However, the combined cross analysis had an increased power of detecting shared QTL by two or more crosses. Indeed, all five significant QTL had a higher LOD score than that achieved in an individual cross. The current and previous B6 × BALB F2 crosses suggested the presence of two QTL on chromosome 5 for carotid atherosclerosis (Rowlan *et al.* 2013a), while the combined cross analysis clearly demonstrated the presence of two disparate QTL on the chromosome. We named the proximal QTL *Cath7* to represent a new locus for carotid atherosclerosis. The significant QTL on distal chromosome 9 identified by the combined cross analysis overlapped with a suggestive QTL previously mapped in the B6 × BALB F2 cross (Rowlan *et al.* 2013a), and was named *Cath8*. Consistent with the conclusion drawn by Li *et al.* (2005), we found that the C.I. defined by the combined cross analysis was smaller than that defined in an individual cross for most of the QTL.

*Cath1* has been mapped in three intercrosses derived from mouse strains, including B6, C3H, and BALB, whose genome sequences are publicly available through the Sanger mouse genomes project. By examining genes containing variants that were shared among the low allele strains (BALB and C3H) but different from those carried by the low allele strain (B6), we reduced the number of candidate genes to 24. Because a QTL is yielded from changes in the function or the quantity of a gene product, we concentrated on genes carrying a nonsynonymous SNP or a SNP in the upstream regulatory region. *Nin*, *Dact1*, and *Rtn1*, which are located underneath the linkage peak and contain one or more nontolerated nonsynonymous SNPs, have shown suggestive associations with increased risk of ischemic stroke (Meschia *et al.* 2011) or lipoprotein particle size (Frazier-Wood *et al.* 2013).

A significant correlation was observed between non-HDL cholesterol levels and atherosclerotic lesion sizes in the present cross. Our previous study of a F2 population also showed a correlation between carotid lesion sizes and non-HDL cholesterol levels (Rowlan *et al.* 2013a). A marginal inverse correlation of HDL cholesterol levels with lesion sizes was observed in this cross, and also in two previous crosses (Li *et al.* 2008; Rowlan *et al.* 2013a). These findings are consistent with the observations made in humans (Mora *et al.* 2007; Sanossian *et al.* 2007). No correlation between carotid lesion sizes and plasma triglyceride levels was observed in this cross, nor in previous crosses (Li *et al.* 2008; Rowlan *et al.* 2013a). We have observed a trend toward a significant correlation of carotid lesion sizes with fasting plasma glucose levels in this cross. Blood glucose levels of the F2 mice were markedly elevated by feeding of a Western diet (Wang *et al.* 2015). In humans, impaired fasting glucose homeostasis has also been associated with preclinical carotid atherosclerosis (De Michele *et al.* 2002).

In summary, we have identified a number of QTL for carotid atherosclerosis, demonstrating the polygenic control of the disorder. The significant correlations of carotid lesion sizes with HDL and non-HDL cholesterol levels suggest that some loci exert effects on carotid atherosclerosis partially through action on lipoproteins. Using bioinformatics tools, we have reduced the list of candidate genes for a major atherosclerosis locus.

## Supplementary Material

Supplemental material is available online at www.g3journal.org/lookup/suppl/doi:10.1534/g3.116.037879//DC1.
