# Supplementary material for: Polygenic Control of Carotid Atherosclerosis in a BALB/cJ × SM/J Intercross and a Combined Cross Involving Multiple Mouse Strains
Source: G3 (Bethesda). 2016 Dec 28;7(2):731–9. doi: 10.1534/g3.116.037879 (PMC5295616; doi:10.1534/g3.116.037879)
Supplement: Supplementary file 1 [file 731FileS1.docx]

File S1: Original genotype and phenotype data used for the current study. (.xlsx, 1 MB)

Available for download as a .xlsx file at:

http://www.g3journal.org/lookup/suppl/doi:10.1534/g3.116.037879/-/DC1/FileS1.xlsx
